# Supplementary material for: Content-rich biological network constructed by mining PubMed abstracts
Source: BMC Bioinformatics. 2004 Oct 8;5:147. doi: 10.1186/1471-2105-5-147 (PMC528731; doi:10.1186/1471-2105-5-147)
Supplement: Additional File 2 — The original results of the above study (non-essential files are deleted to keep the file size under the limit set by BMC bioinformatics). [file 1471-2105-5-147-S2.bz2 › chilibotAdditionalFile2/dip05/399ID9662041E9953/html/EGFR_BTC.html]

 


 **EGFR** and **BTC** 
  
Found 65 abstracts in PubMed, retrieved 05.  
 

 What does Google say? 
 PDF only 
| .edu only 

---

**Interactive relationship** (e.g. stimulation, inhibition, etc)

**Neutral relationship**- Heparin binding epidermal growth factor HB EGF and betacellulin  [ **BTC** ]   **BTC**  are activating ligands for EGF receptor  **EGFR**  ErbB1  [ **EGFR** ]  and ErbB4.  Ref: 12773386 EMBO J< MedlineTA>EMBO J,
- Betacellulin  [ **BTC** ]  and heregulin NDF alpha strikingly colocalized with  **EGFr**  and erbB3 receptors in the granular layer and in a declining gradient from the granular zone to the basal layer, respectively.  Ref: 12768307 Arch Dermatol Res< MedlineTA>Arch Dermatol Res,

**Non-interactive relationship** (e.g. studied together, co-existance, homology, etc.)

- Moreover, immunohistochemical staining revealed that that  **BTC** , ErbB1  [ **EGFR** ] , and ErbB4 are upregulated in the plaques of human atherosclerotic coronary arteries.  Ref: 12869389 Circ ResCirc Res,
- **BTC**  induced phosphorylation of all four EGF receptors present on HTASMCs ErbB1  [ **EGFR** ] , ErbB2, ErbB3, and ErbB4.  Ref: 12869389 Circ ResCirc Res,
